# Survey of Physicians on the Need for Female Chaperones Based on the Body Areas Examined in Female Patients by Male Physicians: A Cross-Sectional Pilot Study

**DOI:** 10.7759/cureus.77208

**Published:** 2025-01-09

**Authors:** Daiki Yokokawa, Takanori Uehara, Rurika Sato, Kosuke Ishizuka, Yu Li, Kiyoshi Shikino, Tomoko Tsukamoto, Hiroki Tamura, Yasutaka Yanagita, Jumpei Kojima, Shiho Yamashita, Masatomi Ikusaka

**Affiliations:** 1 General Medicine, Chiba University Hospital, Chiba, JPN; 2 General Medicine, Yokohama City University School of Medicine, Yokohama, JPN; 3 Community-Oriented Medical Education, Chiba University Graduate School of Medicine, Chiba, JPN

**Keywords:** chaperones, intimate examination, intimate parts, physical examination, physicians' perceptions

## Abstract

Background

In the United States (US), most physicians who commit sexual misconduct are male, and such misconduct is associated with the absence of chaperones. Several organizations recommend a chaperone during all intimate examinations (breast, genital, and rectal exams). However, in Japan, guidelines are not clearly defined, and hospitals and medical societies have not established standardized protocols. At Chiba University Hospital’s Department of General Medicine, female nurses are requested to act as chaperones during female patients’ physical examinations. However, limited medical resources make this challenging. Thus, it is necessary to investigate the necessity of female chaperones for the examination area to prioritize their presence. This study surveyed physicians to determine which examination areas require the presence of female chaperones during the examination of female patients by male physicians. The necessity was stratified and compared by the physician’s sex and age.

Methods

This pilot cross-sectional study surveyed physicians in the Department of General Medicine at Chiba University Hospital. The study content was explained via email, and only those who consented to participate were asked to complete the questionnaire. Only physicians who had passed the Japanese medical licensing examination and had completed two years of residency were included. The primary factors were the physician’s sex and age. The necessity for female chaperones was measured using a five-point Likert scale for different examination areas and patient age groups. The Mann-Whitney U and Kruskal-Wallis tests were also employed.

Results

Responses were obtained from 17 of the 19 physicians (89%; 10 male and seven female). Regardless of sex, there was consensus on the necessity of female chaperones when examining intimate parts (chest, thighs (disrobed), breasts, inguinal region, perineum, and buttocks). Female physicians were more likely to consider chaperones necessary for additional areas, including the head/face (p=0.014), chest (clothed) (p=0.019), abdomen (clothed/disrobed) (p=0.003, 0.033), back (clothed) (p=0.001), buttocks (clothed) (p=0.023), shoulder-upper arm (clothed) (p=0.005), and thighs (clothed) (p=0.033). The necessity for chaperones decreased as the patient’s age increased.

Conclusion

Female physicians presented more cautious opinions, considering chaperones necessary for a broader range of examination areas beyond the traditionally defined intimate parts.

## Introduction

The American College of Obstetricians and Gynecologists, the American Medical Association, the United Kingdom (UK) General Medical Council, the Royal College of Obstetricians and Gynecologists, and the Royal College of Pediatrics and Child Health all recommend the presence of a chaperone for all breast, genital, and rectal examinations (intimate examinations), regardless of the gender concordance between the patient and the physician [1-5]. From a medical safety perspective, a chaperone is necessary to protect both the patient and the physician [6]. A chaperone is an impartial observer present with the patient and the physician during the examination. Typically, they need to be healthcare professionals who respect patient dignity and confidentiality, notice signs of patient discomfort or distress, provide reassurance, are familiar with examination procedures, continuously observe the physician’s conduct, and express concerns about the physician’s behavior or actions, if necessary [3].

In the United States (US), the majority of physicians committing sexual misconduct are male, and such misconduct is linked to the absence of a chaperone [7-9]. Despite recommendations from various medical societies, patients do not always require a chaperone’s presence when the physician and patient are of the same gender. In the US, 60% of female and 70% of male patients do not mind the absence of a chaperone when the physician is of the same gender [10]. Additionally, female patients undergoing pelvic examinations rarely desire a chaperone if the examiner is female [11]. Across all age groups, female patients are more favorable toward the presence of a chaperone than male patients [10].

As of the end of 2020, female physicians in Japan accounted for 22.8% of the population, making it inevitable that male physicians would examine female patients [12]. Intimate part examinations are also necessary for primary care [13]. In the US, higher rates of sexual misconduct complaints occur in family medicine, obstetrics and gynecology, and psychiatry, indicating the need for chaperones in all medical settings [1]. However, in Japan, there are no explicit guidelines or recommendations from municipalities or medical societies regarding the presence of chaperones, leaving the decision to individual medical institutions, with no reports investigating the actual situation.

In the Department of General Medicine at Chiba University Hospital, male physicians must have female nurses as chaperones during the physical examination of female patients, regardless of the examination area. A chaperone is not required if a female physician is part of the examination team. Nurses are the most common chaperones, with 78% of general practitioners in the United Kingdom likely to use nurses as chaperones [14]. Other physicians and trainees are also considered potential chaperones [8,14]. However, the potential for misconduct remains if the chaperone does not meet the required conditions, such as merely being present while performing other clerical tasks or intentionally not observing [8]. Due to the time constraints required for chaperones, having female chaperones present during all examinations under limited medical resources is difficult.

Previous studies investigating the necessity of chaperones when examining areas other than the intimate parts are limited. About 10% of female and 2% of male patients requested chaperones for chest examinations of the heart and lungs, as well as abdominal examinations, in addition to intimate parts [10]. In pediatric outpatient clinics, even visual chest inspections were considered private examinations [15]. Additionally, histories of menstruation, urination, and sexual matters were also recognized as private inquiries [15]. Intimate parts are generally defined as body parts habitually covered by clothing in public places due to fashion or cultural norms, encompassing areas such as the buttocks, groin, anus, perineum, external genitalia, and breasts; however, definitions can vary based on culture and religion. In Japan, no clear medical or official definitions exist. Under Japanese criminal law, “obscene materials” are defined as those that arouse or stimulate sexual desire, harm the usual sense of sexual shame in ordinary people, and violate good sexual moral concepts, but the criteria are ambiguous [16].

No previous studies have investigated the physicians’ perspectives on the necessity of chaperones for specific body areas under examination. There is also a need to investigate areas other than these intimate parts. Therefore, this pilot study aimed to survey physicians regarding the conditions and examination areas for which male physicians consider female chaperones necessary during the physical examination of female patients. Additionally, necessity was stratified and compared according to the physicians’ age and sex.

## Materials and methods

Design, participants, and setting

This was a cross-sectional pilot study using a questionnaire. The survey targeted all 19 physicians affiliated with the Department of General Medicine at Chiba University Hospital as of October 2022. The study content was explained via email, and only those who consented to participate were asked to complete the questionnaire. Only physicians who had passed the Japanese medical licensing examination and had completed two years of residency were included. Physicians who did not provide informed consent were excluded from the study. As this was a pilot study, sample size calculations were not performed.

Data collection

An anonymous questionnaire was administered using Microsoft Forms (Microsoft Corp., Redmond, US). Regardless of the respondent’s gender, they were asked to assume the scenario of “a male physician examining a female patient aged 16 or older in an outpatient setting” and rate the necessity of a female chaperone for each examination area using a five-point Likert scale. Examination areas covered by clothing were divided into disrobed and clothed scenarios. Additionally, areas requiring only visual inspection and those requiring palpation were assessed separately. The questionnaire clarified that visual inspection would be performed when disrobed, and percussion or palpation would be performed when clothed.

Respondents’ gender and age were also collected. The need for chaperones in patients stratified by age was also investigated. The age groups were defined as minors (under 18), young adults (18-39), middle-aged/older adults (40-64), elderly (65-79), and very elderly (80 and older). Additionally, respondents were asked whether a chaperone would still be necessary if there was an accompanying person in the examination room, stratified by the attributes of the accompanying person: female physician, female student, male relative, female relative, male nonrelative, and female nonrelative.

Data analysis

The primary factors were the physicians’ sex and age (20s, 30s, 40s, or older). The primary outcome measures were the necessity of a female chaperone for each examination area, the patient’s age, and the attributes of the chaperone. Statistical analysis was performed using the Mann-Whitney U and Kruskal-Wallis tests using IBM SPSS Statistics for Windows (version 22.0; IBM Corp., Armonk, US).

Ethics statement

This study was conducted as a pilot study based on a survey aimed at operational improvement among physicians. Since the survey was not intended to involve clinical interventions or patient data and falls outside the scope of Japan’s Clinical Trials Act, formal ethical review was not required. The authors determined that ethics committee approval was unnecessary. All procedures adhered to the ethical guidelines established by the Declaration of Helsinki and institutional standards.

Informed consent

The survey was initially conducted for operational improvement purposes, and informed consent was later obtained from all participants to use the collected data for research. Consent was verbally obtained simultaneously with the completion of the survey, with DY as the person responsible for obtaining consent and TU as the witness. Each physician received an explanation of the study’s objectives, and consent for the use of their survey data for research and publication was explicitly obtained. Only adult participants were included in the study, and the confidentiality and anonymity of participants were ensured throughout the process.

## Results

The survey was conducted in October 2022, and responses were received from 17 physicians (89% response rate; 10 male and seven female).

**Figure 1:**
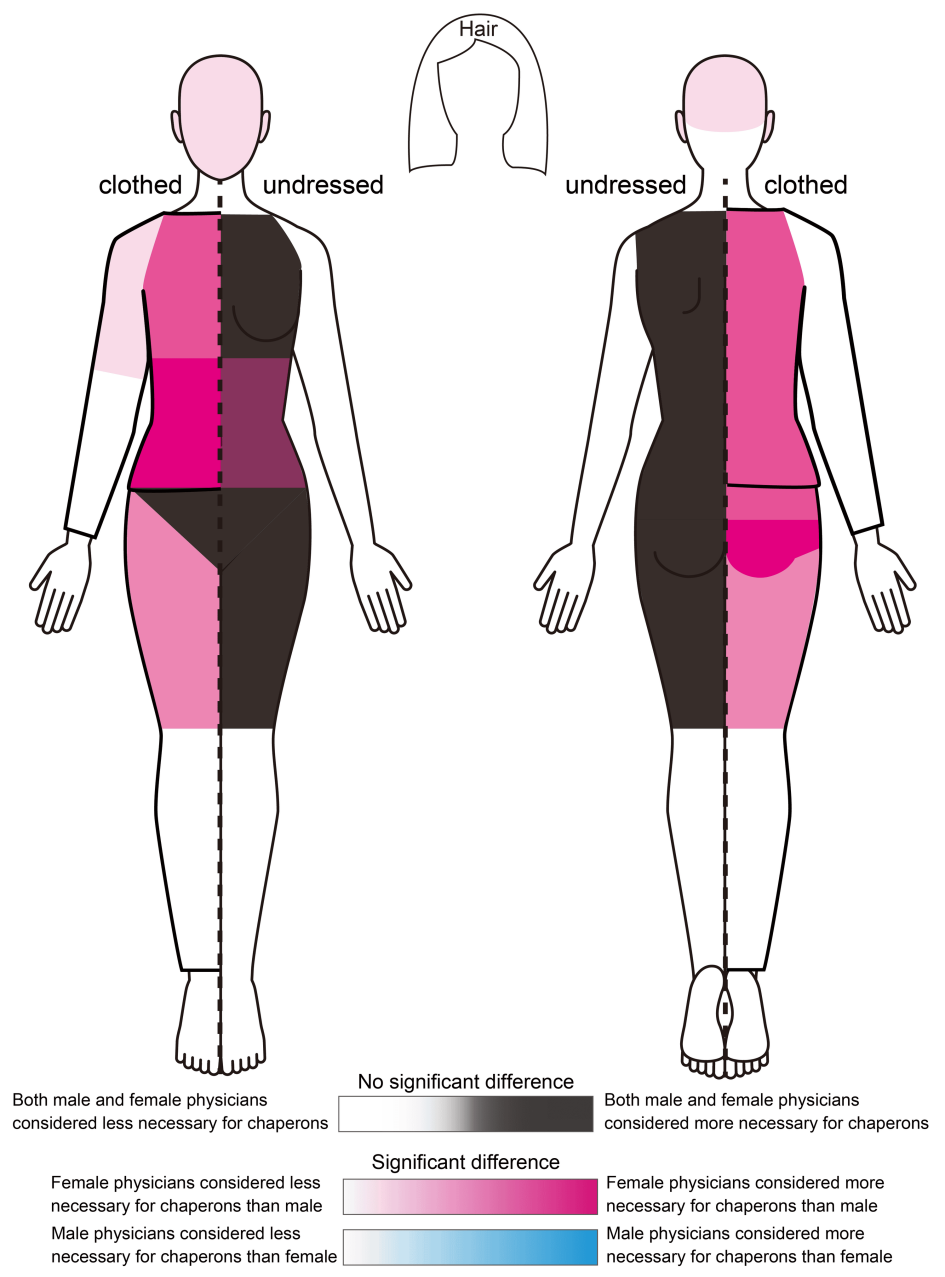
Necessity of chaperones based on physicians’ gender for each examination area The necessity of chaperones by examination area was compared based on the sex of the responding physicians. All physicians responded based on the scenario of a male physician examining a female patient, regardless of sex. The areas shown in monochrome indicate no significant differences between the sexes. Areas shaded in red indicate regions for which female physicians found chaperones more necessary than male physicians; the darker the color, the higher the median necessity for female physicians. The blue gradient indicates areas for which male physicians found chaperones to be more necessary than female physicians. However, no such areas were identified. The figure was created by the authors.

Table 1 presents the results, and Figure 1 illustrates the necessity of chaperones for the examination areas. Regardless of the respondent physician’s sex, there was a high consensus on the necessity of chaperones to examine the chest, back, thighs (all disrobed), breasts, inguinal region, perineum, and buttocks (black areas in Figure 1). Comparatively, female physicians were significantly more likely to consider chaperones necessary for the head/face (p=0.014), chest (clothed) (p=0.019), abdomen (clothed) (p=0.003), abdomen (disrobed) (p=0.033), shoulder-upper arm (clothed) (p=0.005), back (clothed) (p=0.001), buttocks (clothed) (p=0.023), and thighs (clothed) (p=0.022) (red areas in Figure 1). The darker red areas indicate a more significant difference in the median responses between male and female physicians, notably for the abdomen (clothed) and buttocks (clothed). There were no areas for which male physicians were significantly more likely to consider chaperones necessary (blue areas in Figure 1). There were no significant differences based on physicians' age.

**Table 1:** Necessity of chaperones based on physicians’ gender for each examination area using five-point Likert scale (5: very necessary, 1: very unnecessary) The necessity of chaperones by examination area was compared using the Mann-Whitney U test based on the sex of the responding physicians. All physicians responded based on the scenario of a male physician examining a female patient, regardless of sex. M: Male physicians, F: Female physicians, U: Mann-Whitney U value, P: P value, Q3-Q1: Interquartile range, *: Significant difference (p < 0.05)

Body Parts	Group	N	Median	Q3-Q1	Mean rank	Sum	U	P
Hair	M	10	1.00	0	7.65	76.50	21.500	0.440
	F	7	2.00	3	10.93	76.50		
Head and face	M	10	1.00	0	6.50	65.00	10.000	0.014*
	F	7	2.00	3	12.57	88.00		
Neck	M	10	1.00	0	7.00	70.00	15.000	0.055
	F	7	2.00	4	11.86	83.00		
Chest - clothed	M	10	2.00	3	6.65	66.50	11.500	0.019*
	F	7	4.00	1	12.36	86.50		
Chest - undressed	M	10	5.00	0	9.20	92.00	33.000	0.887
	F	7	5.00	1	8.70	61.00		
Abdomen - clothed	M	10	1.50	2	6.10	61.00	6.000	0.003*
	F	7	4.00	1	13.14	92.00		
Abdomen - undressed	M	10	4.00	3	6.80	68.00	13.000	0.033*
	F	7	5.00	1	12.14	85.00		
Breast - undressed	M	9	5.00	0	8.11	66.50	28.000	0.758
	F	7	5.00	0	9.00	86.50		
Groin - clothed	M	8	4.00	3	6.56	52.50	16.500	0.189
	F	7	5.00	1	9.64	67.50		
Groin - undressed	M	9	5.00	1	7.33	61.00	21.000	0.299
	F	7	5.00	0	10.00	92.00		
Perineum - clothed	M	8	5.00	1	6.69	53.50	17.500	0.232
	F	7	5.00	0	9.50	66.50		
Perineum - undressed	M	9	5.00	0	8.11	73.00	28.000	0.758
	F	7	5.00	0	9.00	63.00		
External genitalia - inspection	M	9	5.00	0	8.11	73.00	28.000	0.758
	F	7	5.00	0	9.00	63.00		
External genitalia - palpation	M	9	5.00	0	8.11	73.00	28.000	0.758
	F	7	5.00	0	9.00	63.00		
Gynecological pelvic examination	M	8	5.00	0	7.56	60.50	24.500	0.694
	F	7	5.00	0	8.50	59.50		
Anus - inspection	M	9	5.00	0	8.11	73.00	28.000	0.758
	F	7	5.00	0	9.00	63.00		
Anus - palpation	M	9	5.00	0	8.11	73.00	28.000	0.758
	F	7	5.00	0	9.00	63.00		
Rectal examination	M	9	5.00	0	8.11	73.00	28.000	0.758
	F	7	5.00	0	9.00	63.00		
Shoulder and arm - clothed	M	10	1.00	0	6.25	62.50	7.500	0.005*
	F	7	2.00	2	12.93	90.50		
Shoulder and arm - undressed	M	10	1.00	2	7.10	71.00	16.000	0.070
	F	7	2.00	2	11.71	82.00		
Forearm and hand - clothed	M	10	1.00	0	8.00	80.00	25.000	0.364
	F	7	1.00	1	10.43	73.00		
Forearm and hand - undressed	M	10	1.00	0	8.35	83.50	28.500	0.536
	F	7	1.00	1	9.93	69.50		
Back - clothed	M	10	2.00	3	5.85	58.50	3.500	0.001*
	F	7	4.00	1	13.50	94.50		
Back - undressed	M	10	5.00	0	8.15	81.50	26.500	0.417*
	F	7	5.00	1	10.21	71.50		
Buttocks - clothed	M	10	1.50	2	6.17	55.50	10.500	0.023*
	F	7	4.00	1	11.50	80.50		
Buttocks - undressed	M	10	4.00	3	7.72	69.50	24.500	0.470
	F	7	5.00	1	9.50	66.50		
Thigh - clothed	M	10	2.50	3	6.80	68.00	13.000	0.033*
	F	7	4.00	1	12.14	85.00		
Thigh - undressed	M	10	4.50	3	8.30	83.00	28.000	03536
	F	7	5.00	1	10.00	70.00		
Lower leg and foot - clothed	M	10	1.00	0	7.50	75.00	20.000	0.161
	F	7	1.00	2	11.14	78.00		
Lower leg and foot - undressed	M	9	1.00	0	6.56	59.00	14.000	0.071
	F	7	2.00	4	11.00	77.00		

**Figure 2:**
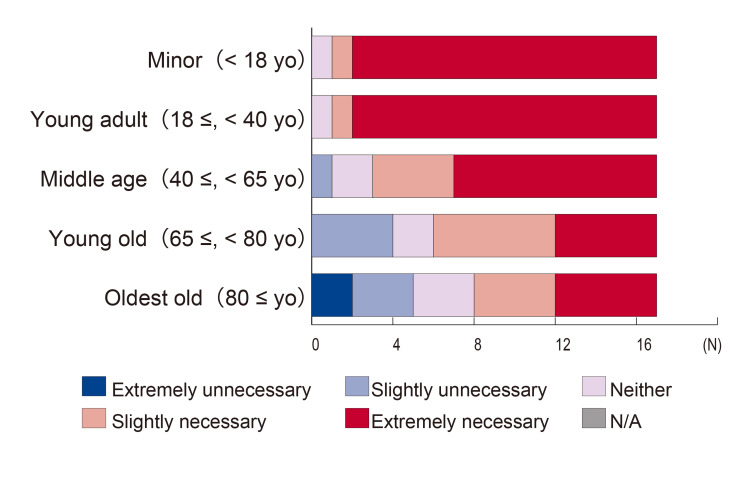
Necessity of chaperones based on patients’ age No significant differences were found based on the sex of the responding physicians. The need for chaperones tended to decrease as the patient's age increased. N/A: Not applicable

Figure 2 shows the necessity of chaperones stratified by patients' age. There were no significant differences based on the gender of the respondent physicians. As the patients’ age increased, more physicians considered chaperones unnecessary. There were no significant differences based on respondent physicians' age.

**Figure 3:**
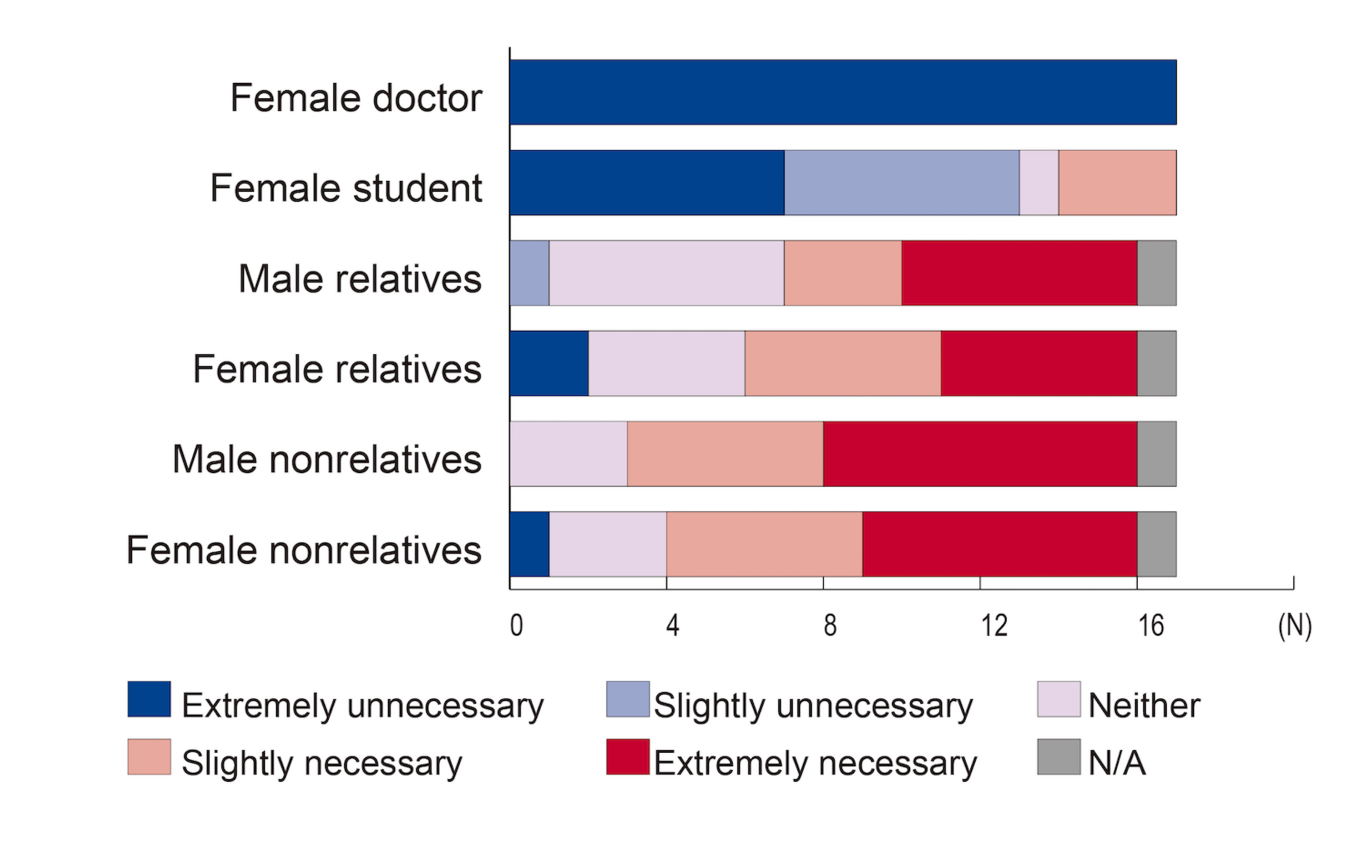
Necessity of chaperones based on the attributes of the accompanying person in the examination room Assuming that a male physician examines a female patient, the necessity of a nurse chaperone was assessed even when another person was present in the room. Physicians and medical students were considered adequate chaperones, whereas relatives and nonrelatives were considered insufficient. N/A: Not applicable

Figure 3 shows whether a nurse chaperone would still be necessary if there was an accompanying person in the examination room, stratified according to the attributes of the accompanying person. When the accompanying person was a female physician or student, most respondents considered a chaperone unnecessary. There were significant differences based on the respondent physician’s gender; female physicians were more likely than male physicians to consider a chaperone necessary even if the accompanying person was a male (median 5.0, 3.0, U=5.500, p=0.003) or female relative (median 5.0, 3.0, U=8.000, p=0.012). There were no significant differences based on physicians' age.

## Discussion

This study is the first to investigate the necessity of chaperones for specific examination areas when male physicians examine female patients by dividing the examination areas in detail. By targeting the physicians who conducted the examinations, this study ensured that the scenarios for each examination area could be easily visualized, allowing for a thorough investigation. Intuitively, both male and female physicians considered chaperones necessary for intimate examinations (black areas in Figure 1) and unnecessary examinations of areas such as the scalp or distal limbs (white areas in Figure 1). However, areas where female physicians considered chaperones necessary more significantly (red areas in Figure 1) can be viewed as broadly defined intimate areas. Prior research and guidelines from other countries regarding the necessity of chaperones for non-intimate partners are limited. For instance, only 27% of the chaperones were used during chest and abdominal examinations [10]. Our study suggests that female physicians perceive a broader range of intimate parts and consider chaperones necessary to protect both the physician and patient, even when the patient is clothed. While it might be assumed that male physicians would prefer chaperones to prevent false accusations of sexual misconduct, our study showed that female physicians were more precautionary. This might be because female physicians who share the same sex as female patients may incorporate the patient’s perspective into their professional opinions.

Regarding the necessity of chaperones based on the patient's age, previous reports support our finding that younger patients motivated the need for chaperones [13,17]. For male physicians, the youth or unmarried status of the patient is a significant factor in deciding to use a chaperone [18]. However, guidelines in the UK and US do not specify the age of patients for chaperone use [1-5]. While it is imaginable that feelings of embarrassment or sexual arousal might change with age and experience, the right to have a chaperone should not diminish with age and should be offered at all ages.

The unavailability of nurses as chaperones (due to human resources shortages) is a barrier to using chaperones [19]. Factors influencing chaperone use include availability, time constraints, confidentiality, and patient factors such as instinct, psychiatric history, and being an ethnic minority [14]. Guidelines state that chaperones should be healthcare professionals, as relatives or friends are not impartial observers and are inappropriate as chaperones [3]. In practice, healthcare professionals’ attitudes and behaviors often do not align with these recommendations, leading to concerns that chaperones are not used according to guidelines [13,14,18,20]. Patients may also be unwilling to accept a chaperone, especially teenagers [10,20-23]. In intimate examinations in family medicine, 68% of male and 48% of female patients do not mind the presence or absence of a chaperone [23]. Some patients even prefer relatives or friends as chaperones, and children and teenagers mainly prefer a parent’s presence over healthcare professionals [10,15,20]. In our study, relatives were considered inadequate as chaperones, likely due to concerns about medical safety.

This study has several limitations. First, as a pilot study, the sample size was small and limited, necessitating the cautious generalization of the results. Second, the scenario was restricted to “male physicians who examined female patients.” Female physicians and male patients have fewer opportunities to use or be assigned chaperones [17]. A 2005 report from the UK indicated that 37% of general practitioners had a policy on chaperone use, and 54% of male physicians always offered chaperones, compared to only 2% of female physicians [14]. The differing perspectives on chaperone use between male and female physicians likely stem from their experiences and gender. Future research should expand the scenarios to include “female physicians examining male patients” and “same-gender physician-patient examinations,” as guidelines state that the need for a chaperone is independent of the patient’s or physician’s gender [3].

## Conclusions

In situations where male physicians conduct physical examinations of female patients, not only the narrow definition of intimate parts - such as breasts, genitals, and anus - but also proximal limbs and abdominal examinations can be considered broadly defined intimate parts, thus necessitating a chaperone. Additionally, the study indicates that female physicians tend to have more cautious views regarding the need for chaperones.
